# Self-Powered Self-Contained Wireless Vibration Synchronous Sensor for Fault Detection

**DOI:** 10.3390/s22062352

**Published:** 2022-03-18

**Authors:** Ghufran Aldawood, Hamzeh Bardaweel

**Affiliations:** 1 Institute for Micromanufacturing, College of Engineering and Science, Louisiana Tech University, Ruston, LA 71272, USA; gja005@latech.edu; 2 Department of Mechanical Engineering, College of Engineering and Science, Louisiana Tech University, Ruston, LA 71272, USA; 3 Department of Nanosystems Engineering, College of Engineering and Science, Louisiana Tech University, Ruston, LA 71272, USA

**Keywords:** vibration energy harvesting, vibration sensor, self-powered sensor, clean technology, wireless vibration sensor, IoT support technology

## Abstract

Failure in dynamic structures poses a pressing need for fault detection systems. Interconnected sensor nodes of wireless sensor networks (WSN) offer a solution by communicating information about their surroundings. Nonetheless, these battery-powered sensors have an immense labor cost and require periodical battery maintenance and replacement. Batteries pose a significant environmental threat that is expected to cause irreversible damage to the ecosystem. We introduce a fully integrated vibration-powered energy harvester sensor system that is interfaced with a custom-developed fault detection app. Vibrations are used to power a radio frequency (RF) transmitter that is integrated with the vibration sensor subunit. The harvester-sensor unit is comprised of dual moving magnets that are bordered by coil windings for power and signal generation. The power generated from the harvester is used to operate the transmitter while the signal generated from the sensor is transmitted as a vibration signal. Transmitted values are streamed into a high precision fault detection app capable of detecting the frequency of vibrations with an error of 1%. The app employs an FFT algorithm on the transmitted data and notifies the user when a threshold vibration level is reached. The total energy consumed by the transmitter is 0.894 µJ at a 3 V operation. The operable acceleration of the system is 0.7 g [m/s^2^] at 5–10.6 Hz.

## 1. Introduction

Internet of Things (IoT) technologies are blooming and are expected to reach a 1567 billion USD market value by 2025 [1]. Currently, there are a little over 7 billion sensor nodes worldwide [1]. These sensors represent the backbone of IoT systems since they are responsible for detecting essential information about the surrounding environment and monitoring the health conditions of structures such as compressors, pumps, bridges, tunnels, railroads, and other dynamic structures [2]. Monitoring the health conditions of structures using these sensors helps in preventing catastrophic failures and loss of lives [2].

Presently, the majority of these sensors are powered using traditional batteries [3]. The use of these traditional batteries limits the scope and value of these IoT sensors [4,5]. This is due to the fact that the use of conventional batteries as a power source for IoT sensors results in several challenges. Not only do these batteries require continuous and frequent replacement and maintenance [6], but also they have a limited lifespan and pose an environmental threat [7]. Given the projected worldwide spread of these IoT sensors, this environmental concern has become a pressing issue to deal with [8]. Moreover, in harsh environments and remote locations, including wildlife, gas and oil fields, and drilling in mining fields, a self-powered solution becomes the only viable option for deploying long-lasting, stand-alone, eco-friendly, and sustainable sensors [9].

Consequently, there has been a growing interest in developing autonomous, self-powered, environment-friendly sensor technologies as a necessary and integral part of the flourishing IoT systems and technologies [10]. Tremendous recent studies have investigated the issue of developing autonomous sensors powered by free sources of energies surrounding these sensors including solar and vibration energies. Vibration-powered sensors are an attractive option since they are not constrained by the availability of sunlight and can operate indoors and underground [3]. Moreover, vibration energy consists of a broadband vibration spectrum rich in low frequencies with a power density as high as 500 µW/cm^3^ [11]. Vibration energy also represents a wealthy form of freely-available energy in transportation [12] and industry sectors [13].

To this end, Xin Li et al. developed a vibration-powered sensor node [3]. The system used a piezoelectric transducer to harvest ambient vibrations. The fully integrated self-powered sensing and transmitting system consisted of a few units including energy generation, energy transduction, energy-boosting, energy management and circuitry, and demonstration unit (mobile interface). The system was successfully demonstrated under various vibrations conditions. In another effort, two wireless sensor nodes were powered using an electromagnetic vibration energy harvesting system [1]. A custom-built power conditioning system was integrated into the energy harvesting system and then used to power a sensor node with a duty cycle of 30 s. The self-powered system was shown to produce enough power to receive and transmit information at intervals of less than 60 s. Moreover, in Ref [14] a piezoelectric energy harvester was used to power a wireless platform which consisted of a vibration sensor, a microcontroller, a power management circuitry, and a custom-built low power radio transmitter. The fully integrated system was operated at acceleration level and frequency of 0.25 g [m/s^2^] and 100 Hz, respectively. The system was able to transmit the sensor data every 10 s for a duty cycle of 0.2%. Moreover, in their study, Lu Wang et al. [15] built a wireless temperature sensor node powered by a piezoelectric bimorph cantilever vibration energy harvesting system. A big proof mass was attached to the harvester to lower its resonant frequency to approximately 22 Hz. A power management circuitry was built for rectifying the output from the harvester which was then used to power the temperature sensor. In Ref [16], the authors utilized a commercial piezoelectric cantilever as an energy harvester for powering a wireless temperature sensor node. The study focused on investigating the design methodology for the power management circuitry used in their work. A demonstration of the self-powered temperature sensor was performed. Additionally, Lu Wang et al. [17] built a hybrid piezoelectric-triboelectric unit and used it to construct an autonomous wireless sensor node where the piezoelectric generator served as the energy source and the triboelectric worked as an accelerometer (i.e., sensing unit). The piezoelectric harvester produced approximately 6.5 [mW] at 1 g [m/s^2^] and 25 [Hz] and the triboelectric accelerometer showed a sensitivity of 15 V/g for acceleration range 0–1.5 g [m/s^2^]. In another effort, a piezoelectric energy harvesting-sensor unit was developed and implemented in monitoring airflow from an HVAC outlet [18]. To avoid signal distortion from the sensor the proposed system used two separate piezoelectric devices (i.e., one for energy harvesting and one for sensing purposes). 

One of the major constraints in wireless sensor technologies is associated with the limitations in the supplied energy to the wireless sensor nodes [19,20,21]. Overcoming this challenge can only be achieved through minimizing energy consumption by means of ultra-low-power techniques. Several techniques to reduce energy consumption have emerged in recent years, such as duty cycling [22]. Other techniques include topology control [23], which deals with the distribution of the wireless sensor nodes in order to reduce energy consumption while eliminating interference at the lowest cost possible. Additionally, data transmission network protocol selection is based on application, where different protocols have variable bandwidths with varying energy consumptions [24]. Cyber security of those networks is also amongst the necessary features that when added would require more processing power from the sensor node [25].

The work presented in this article is focused on developing a novel, self-powered, self-contained, environment-friendly, and wireless vibration sensor. One of the major issues that the vibration-powered wireless sensor node designers are faced with is the unstable power source causing much noise to the transmitted data [14]. This causes a need for stringent supply regulation using linear or switching regulators that amounts to power losses due to heat as a result of the regulation. In this work, power is conserved from not using any regulators, and instead, the noise corrupted data is retrieved from post-processing through a custom-developed dynamic fast Fourier transform (FFT) app. A given dynamic structure has a vibration signature where a frequency shift can be detected by implementing an FFT algorithm to the time series data [26]. Furthermore, there is an ample amount of noise introduced into the signal due to interference from the use of radiofrequency modules [14] and the use of FFT in post-processing of the data lessens the impact of noise on the signal [27]. The custom developed app in this work can fetch the sensor bit-stream, buffer the data, and use it to plot the vibration signal amplitude and its frequency in real-time.

Moreover, unlike in the relatively high voltages produced in piezoelectric transducers that would set a need for voltage regulation [3], in this work, electromagnetic transduction is used and the output voltages are within the electronics acceptable supplied voltage range. Also, unlike the aforementioned studies and state-of-the-art developments, in this work, the self-powered sensor uses vibrations to synchronously perform two functions. First, these free vibrations are converted into useful electric power through the presented energy harvester system. Second, these vibrations are detected as electric signals (voltage) by the presented vibration sensor, and are then transmitted wirelessly to the workstation (laptop). Thus, the harvester-sensor hardware is self-contained and self-powered. That is, the mechanical power required to operate the sensor is obtained from the energy harvester. A charge pump circuit, also known as a voltage multiplier, is used to rectify the AC output of the energy harvester. The output DC is then stored in a supercapacitor that provides the energy to the microcontroller and sensor transmitter circuit. The voltage multiplier circuit allows immediate circuit startup due to the transmitter circuit having sufficient voltage and electric current to operate. The signal from the sensor is sampled using a 10-bit analog to digital converter (ADC) and is transmitted over an RF amplitude modulated (AM) carrier. When monitoring the health conditions of a structure, a shift in its vibrations signature may indicate a malfunction in the structure which could lead to impending failure [2,28]. In this article, collected sensor data are analyzed through a custom-developed dynamic displacement monitoring software which helps in mitigating damage to vibrating structures.

The structure and organization of the article are outlined next. The design and structure of the self-powered self-contained wireless sensor are presented in Section 2. The manufacturing and fabrication of the system components are detailed in Section 3. Experimental methods and testing techniques are detailed in Section 4. Results and findings from this work and system operation and demonstration are discussed in Section 5. Finally, Section 6 presents the major conclusions and summarizes the results from this work.

## 2. Design Concept and System Configuration

**Figure 1:**
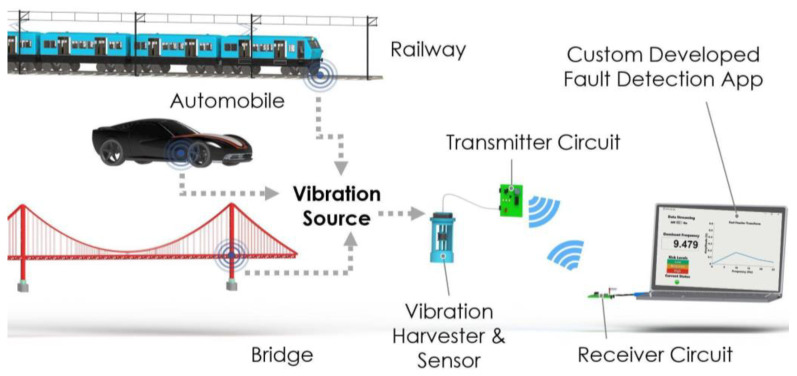
Design and concept of the wireless self-powered, self-contained, and eco-friendly vibration sensor system and its main components.

**Figure 2:**
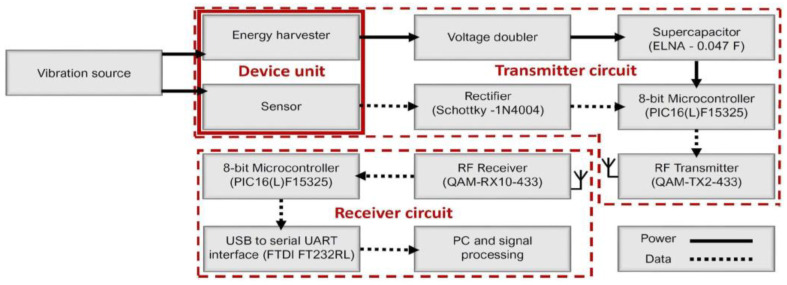
Block diagram showing the layout of the proposed wireless self-powered, self-contained, eco-friendly vibration sensor system.

The concept and overall structure of the proposed self-powered self-contained fully integrated system are shown in Figure 1. The overall structure of the self-powered sensor consists of a few main sub-systems, namely a vibration energy harvester-sensor unit, a transmitter circuit, a receiver circuit, and a custom-developed fault detection app. The main elements and components of these sub-systems are shown in Figure 2.

### 2.1. Vibration Energy Harvester-Sensor Subsystem

**Figure 3:**
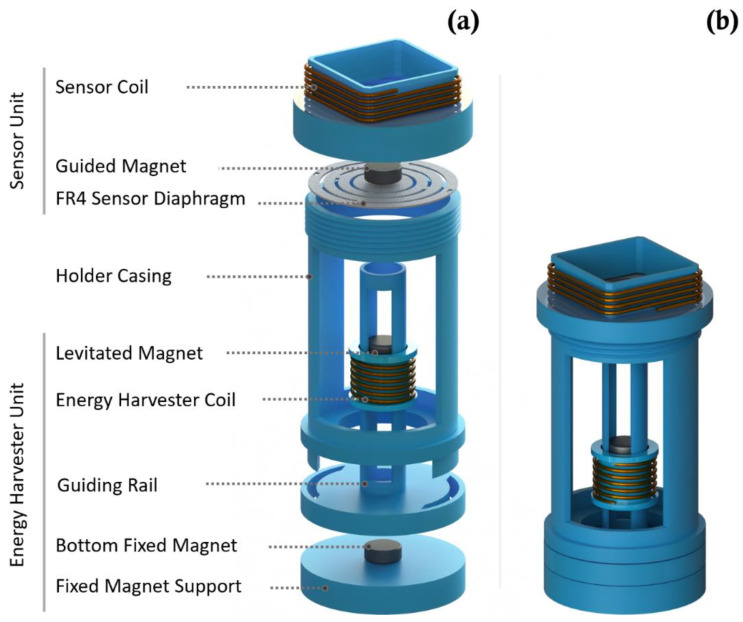
Design and 3D view of the structure of the vibration energy harvester-sensor unit presented in this work: (**a**) exploded view and (**b**) collapsed view.

The vibration harvester-sensor unit consists of two major components: a vibration energy harvester and a vibration sensor (as shown in Figure 3). The harvester consists of two (top and bottom) magnets with a third magnet that is levitated between them. The magnets are arranged in a repulsive configuration with alike poles facing each other and, therefore, the levitated magnet is floating between the top and bottom magnets. The bottom magnet is fixed while the top magnet is glued to, and guided by, a mechanical FR4 sensor diaphragm. A 40 AWG stationary copper coil is wound around the levitated magnet for electric power generation. The vibration sensor consists of the mechanical diaphragm and its guided top magnet, and copper coil windings are positioned around the top magnet as shown in Figure 3. The 3D printed guiding rail of the levitated magnet is designed to provide a restricted travel pathway for the levitated magnet between the top guided magnet and the bottom fixed magnet. The energy harvester coil windings are wound around fixed cylindrical support that is positioned in alignment with the center of the levitated magnet. The fixed magnet support is used to hold the bottom magnet to the guiding rail frame. The 3D printed holder casing is designed to hold the guiding rail of the levitated magnet to the rest of the components as shown in Figure 3.

When subject to external vibrations, first, the levitated magnet moves inside the harvester’s coil windings, thus converting the kinetic energy from these oscillations into electric power that is used to operate the system shown in Figure 1. Consequently, dynamic displacement is induced in the sensor diaphragm and top magnet as a result of these excitations. In turn, induced vibrations in the sensor’s diaphragm and top magnet result in induced voltage in the top coil surrounding the top guided magnet. The voltage signal from the top coil is then sampled by the microcontroller in the transmitter circuit. This is discussed in further detail next. 

### 2.2. Transmitter Subsystem

**Figure 4:**
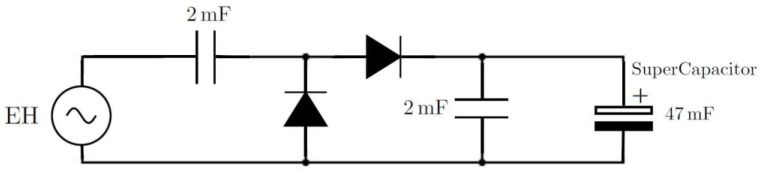
Circuit diagram of the voltage doubler used to rectify the input AC output voltage from the energy harvester.

The main components of the transmitter sub-system are shown in Figure 2. The circuitry encapsulates a Microchip Technology PIC microcontroller that is enabled when the energy harvester has sufficient energy to power the circuit load. The input AC voltage from the energy harvester is rectified by a two-stage voltage multiplier, known as ‘voltage doubler’. This is shown in Figure 4. In the voltage doubler circuit, the diode in the first stage of the doubler is forward biased during the negative half cycle of the input sinusoidal waveform. This allows charging up of both capacitors. Meanwhile, during the positive half cycle of the input, the diode in the first stage of the multiplier is reverse biased and is blocking the discharging of the capacitor in the first stage. This allows for the capacitor on the second stage to charge up to approximately twice the voltage of the input source voltage. The output DC voltage from the voltage doubler is stored in the supercapacitor to power the load.

The energy buffer for the system load is chosen to be a supercapacitor, due to being maintenance-free as well as for its long lifetime and superior power density over chemical batteries [29]. The microcontroller draws the stored energy in the supercapacitor and uses it to power the system while allowing the supercapacitor to recharge in between the transmission cycles. An RF solutions radio frequency transmitter/receiver module with a high operating voltage range is used for the vibration sensor packetized data ahead of post-processing by the custom-developed app. To allow for minimum energy consumption and constant power savings, when vibration energy falls below a threshold value, the microcontroller is set to go into idle mode. Similar to sleep mode, in idle mode, the CPU clock is turned off. However, in idle mode, the microcontroller peripheral clock stays on.

### 2.3. Receiver Subsystem

As shown in Figure 2, the receiver subsystem is composed of the RF receiver, an 8-bit microcontroller, USB to serial UART interface, and the custom-developed dynamic FFT app. The AM radio frequency receiver can receive the transmitted data at a range of 50 m. The microcontroller receives the bit-stream through a universal serial bus (USB) to transistor-transistor logic (TTL) interface at a rate of 300 baud. The received data packet is composed of 1 start bit, 8 data frame bits, and 1 stop bit. The data sampled from vibrations is then run through the in-house custom-made dynamic spectrum analysis FFT app. In the app, the user is prompted to turn data streaming on or off. Once the streaming is turned on, the app stores both voltage and time data into an equal size window buffer. FFT algorithm is carried out on the buffered vibration data, and the output vibration and amplitude of the signal are plotted in real-time on the graphical user interface (GUI) axes. A frequency tracking numeric field feature is also integrated into the app to offer a more distinguishable frequency monitoring.

## 3. Manufacturing and Prototyping

This section describes the details of manufacturing, integration, and assembly of the hardware of the self-powered sensor presented in this work. Additionally, details of signal acquisition and data post-processing via the custom-developed fault detection MATLAB app are discussed in this section.

### 3.1. Fabrication and Integration of System Hardware

**Figure 5:**
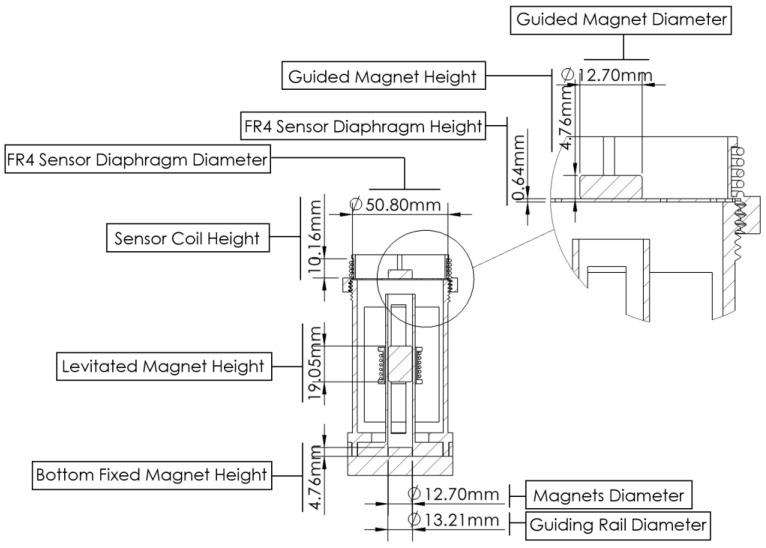
Cross-section view of the vibration energy harvester-sensor unit along with a blow-up detailed section view.

**Figure 6:**
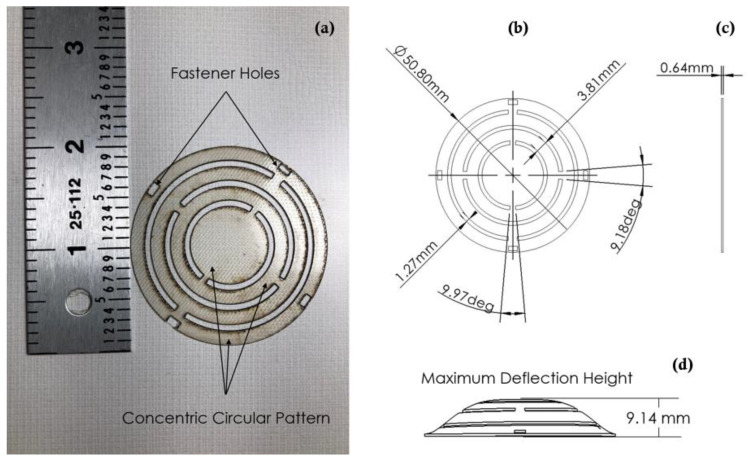
Design and fabrication of the FR4 Sensor diaphragm: (**a**) Fabricated sensor diaphragm next to a scale, (**b**) CAD model top view and dimensions of the sensor diaphragm, (**c**) CAD model side view of the sensor diaphragm and, and (**d**) simulated CAD view of the deflected sensor diaphragm.

**Figure 7:**
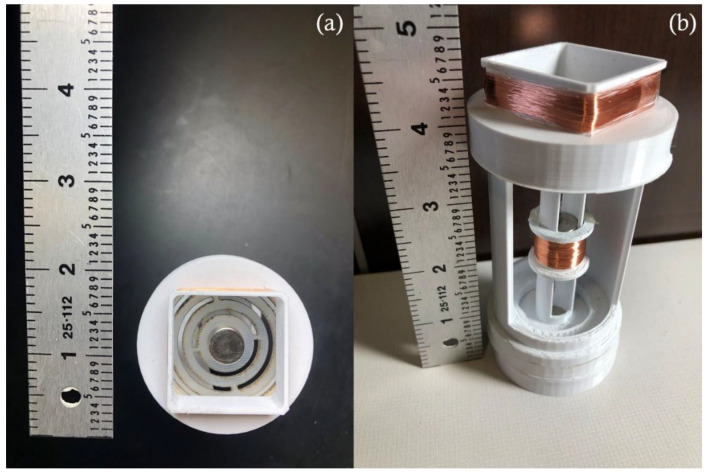
Fully assembled energy harvester-sensor unit next to a scale: (**a**) top view of the unit and (**b**) side view of the unit.

The energy harvester-sensor unit was designed using a 3D CAD designing software (SolidWorks). The casing of the energy harvester was printed using a 3D printer and polylactic acid (PLA) filament. The FR4 sensor diaphragm customized patterns were fabricated using a Kern laser cutter (KER4824-Ti100 micro). The 130 W laser cutter was set at 80% of full power and a cutting rate of 20 mm/s. Three permanent solid magnets were used in the device assembly. The bottom magnet was fixed to the bottom support, the top magnet was fixed to the FR4 sensor diaphragm, and the levitated magnet was left to float. The levitated magnet was guided by the walls of the guiding rail tube as shown in Figure 3b. A manual winding machine (MXBAOHENG NZ-1) was used to wind the enameled copper coils around the harvester and sensor. Details and dimensions of the designed and fabricated structures are shown in Figure 5 and Figure 6. A view of the final assembled energy harvester-sensor unit is shown in Figure 7. A list of the design specification and materials used to fabricate the energy harvester-sensor unit is shown in Table 1.

**Figure 8:**
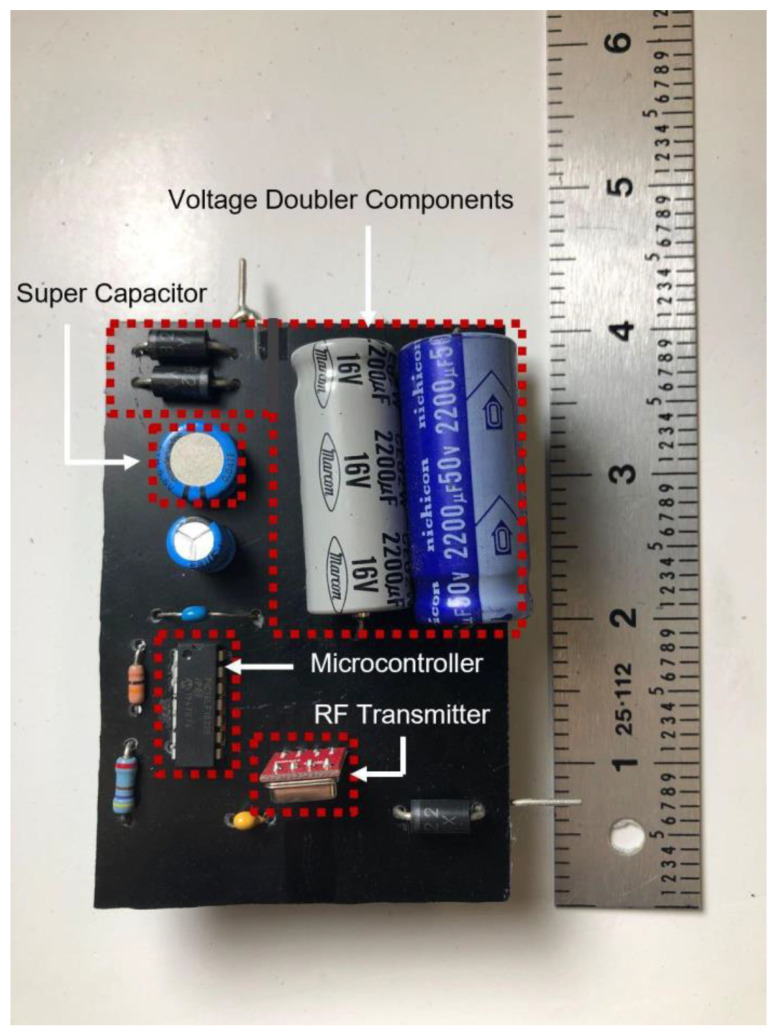
Circuit and components of the transmitter subsystem as part of the manufacturing process of the overall wireless sensor system presented in this work.

The transmitter circuit electronic components, shown in Figure 8, were affixed on an insulating board using soldering. The PIC microcontroller that was chosen (PIC16LF15325/45) had ultra-low-power features where it typically consumed only 8 µA at 32 kHz oscillator frequency and 50 nA at 1.8 V in sleep mode. The PIC also included a windowed watchdog timer feature that was able to issue a reset to the microcontroller in the event of software failure. Furthermore, the microcontroller peripheral module disable (PMD) feature was used to disable all of the unused peripherals to minimize power consumption.

**Figure 9:**
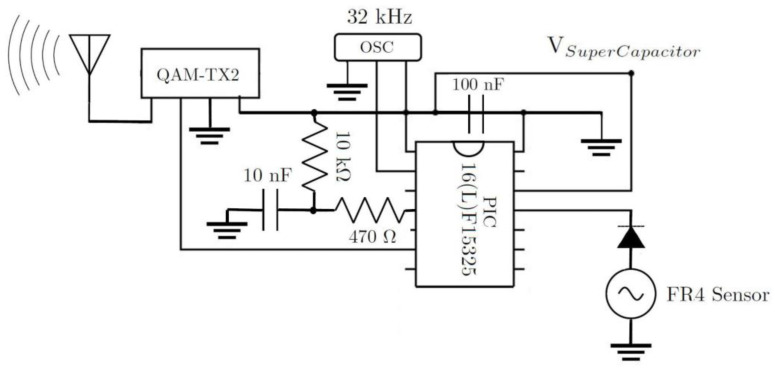
Circuit diagram and interconnections of the microcontroller transmitter subsystem components used in the wireless vibration sensor system presented in this work.

Other components on the circuit board included a 433 MHz RF transmitter module, voltage doubler components incorporating two 16 V, 2200 µF electrolytic capacitors, and two Schottky diodes. The circuit also holds a supercapacitor energy buffer size 47 mF with a 5.5 V voltage rating and a low equivalent series resistance (ESR) of 25 Ω. A circuit diagram and interconnections of the transmitter circuit board are shown in Figure 9.

### 3.2. Signal Acquisition

**Figure 10:**
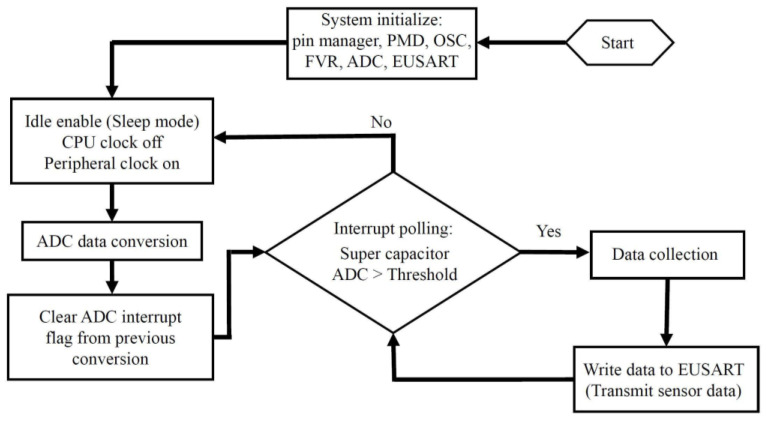
A signal acquisition process flowchart of the wireless vibration sensor presented in this work showing detailed description of the program starting at the initialization process of the microcontroller and ending at the transmission of data.

Both microcontrollers used in the transmitter and receiver subsystems were programmed through the PIC embedded applications development freeware (MPLAB X IDE) and a PICkit 3 in-circuit debugger. The vibration signal acquisition process described in Figure 10 starts when the microcontroller exits the idle (sleep) mode. The microcontroller is programmed to stay in sleep mode until it is prompted to ‘wake up’ when triggered by the voltage level held by the supercapacitor. The microcontroller waking up process is set up to initiate when the sampled signal from the supercapacitor reaches (2 V). The supercapacitor voltage signal sampling takes place at one of the 10-bit low power successive approximation ADC channels of the microcontroller (ADC-CH1). The ADC allows conversion of the analog signal into a 10-bit binary form of that input signal. The ADC channel input voltage level can vary from 0 V up to a maximum voltage that needs to be set as the reference voltage of the channel. A reference voltage is needed by the ADC to create a range of voltages that are mapped into specific length binary values. The supercapacitor sampled input voltage is then compared to those binary values from the reference. Typically, the reference voltage used for ADC channels is the supply voltage to the microcontroller. This poses a challenge since the supply voltage and the sampled voltage would both be of the supercapacitor voltage. This was overcome by using the microcontroller internal fixed voltage reference (FVR) feature that is independent of the microcontroller supply voltage. A programmable independent buffer gain amplifier is used at the output of the FVR and is set to amplify the voltage reference to a desired selectable voltage level. During sampling, the supercapacitor voltage is compared to the FVR and when the value returned matches the selected voltage level, power is delivered to the rest of the system after waking the microcontroller up. During the microcontroller system initialization process, voltage rails are stabilized, and the CPU starts fetching code instructions and data to operate the necessary control registers.

Embedded systems interrupts are hardware features that preempt normal program code operation in order to execute a command that requires CPU attention. As shown in Figure 10, when an interrupt is set, an interrupt service routine firmware (ISR) determines the source of the interrupt by the process of polling. The ISR polling protocol is an active process of monitoring interrupt flag bits from the interrupt flag register. The peripheral interrupt from the supercapacitor voltage level value reaching the set threshold allows the CPU to service that interrupt and wake the microcontroller from sleep. As indicated in Figure 10, unless the supercapacitor voltage level is above the preset threshold, the ISR will continue polling and remain in sleep mode. A command to clear the interrupt flag from a previous interrupt is necessary to execute following the servicing of an interrupt. If the interrupt flag is not cleared, and if the supercapacitor voltage level is above the preset threshold, interrupts will occur repeatedly overriding necessary CPU functions. 

In idle mode the microcontroller CPU core and memory operations are halted while the internal peripheral clocks such as ADC channels clock continue to operate. Once the microcontroller is woken up, the sensor data are sampled through a second ADC channel (ADC-CH2). In Figure 10, the ADC logs the sensor data and writes it to the enhanced universal synchronous and asynchronous receiver transmitter (EUSART) register upon waking up from idle mode. The data are then wirelessly transmitted via the 433 MHz AM RF transmitter module. The signal is then received by a compatible receiver module (QAM-RX10) that is connected to a second microcontroller in order to receive the data through the serial port of a PC by utilizing a USB to serial converter. The antenna-equipped receiver provides a two-way communication that transforms the electromagnetic waves into electrical signals. Modulation of the baseband data onto the carrier is accomplished by amplitude shift keying (ASK) of the signal.

### 3.3. Custom Developed Fault Detection MATLAB App

In this work, the monitoring app is developed using MATLAB-GUI to extract useful information from the collected vibration signal including maximum displacement amplitude and frequency. The extracted information is then compared against preset threshold values to assess the risk level associated with the operation performed.

**Figure 11:**
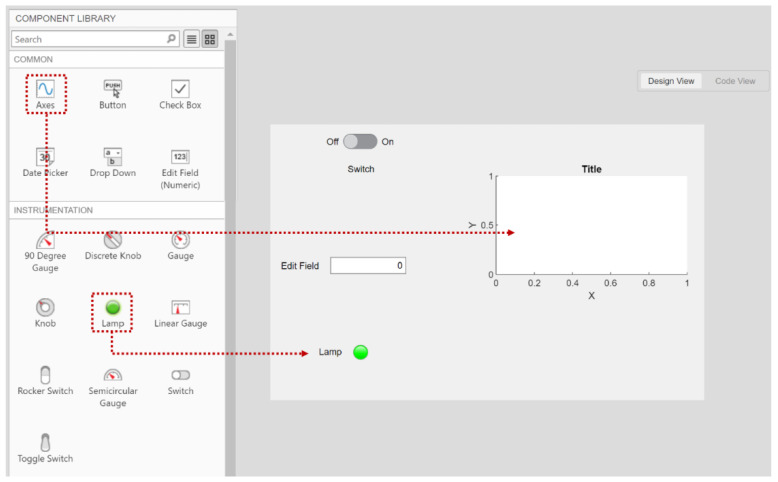
The design view browser inside MATLAB app development environment. The dynamic objects are chosen from a component library and are populated into the GUI canvas.

Inside the MATLAB app development environment, the GUI components including the data streaming switch, frequency tracking numeric field, status indicator, and real time FFT plot are identified as dynamic objects. The objects are chosen from a MATLAB supported components library as seen in Figure 11. The dynamic objects are configured as public access properties that allow data exposure to the user through the GUI. The corresponding values to the communication port and baud rate of the receiver board are then specified. This allows for initiation of data streaming through the serial port when the ‘on’ dynamic object under the data streaming label is selected by the user. Evenly sized sectioned data buffers are set up for both voltage and time elements to allocate for real time data plotting. Parameters of the FFT measurements including the length of the signal, sampling frequency, and Nyquist frequency are identified to convert the windowed time domain data into frequency domain. A peak finder function is then used to detect the dominant frequency from the converted data for display in the GUI numeric field region. The amplitude of the resultant peak is then compared against fixed threshold values to vary the color of the status lamp indicator on the GUI. The variable colors of the lamp give the user a risk severity measure of the performed operation. Therefore, the indicator lamp switches color from green when the energy harvester sensor unit is subject to low amplitude vibrations to red once it experiences higher vibration amplitudes. Further details about the custom developed fault detection MATLAB app are discussed later in this article (i.e., Section 5).

## 4. Experimental Methods and Characterization Techniques

**Figure 12:**
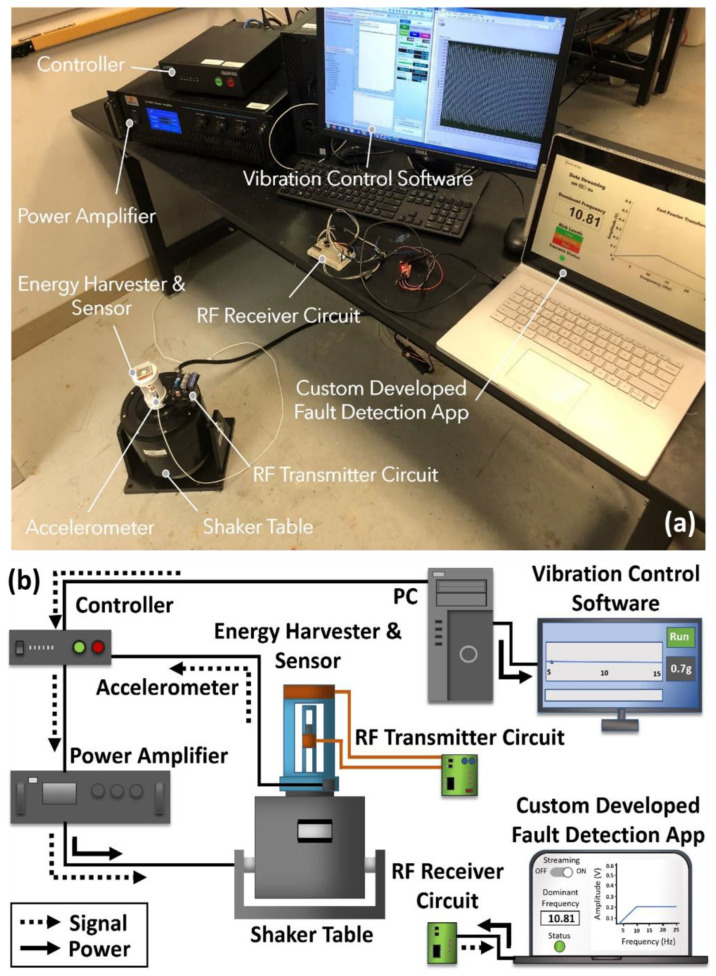
Experimental methods used in this work: (**a**) experimental apparatus used for characterization of the wireless self-powered vibration sensor system presented in this work; (**b**) cartoon schematic of the characterization setup showing signal and power flow in the equipment.

The experimental testing setup shown in Figure 12a was used to measure the dynamic frequency response of the wireless sensor system presented in this work. An illustration of the signal and power flow of the experimental testing equipment setup is shown in Figure 12b. In the closed loop vibration testing system, as shown in Figure 12a, a vibration controller (S81B-P02, SENTEK DYNAMICS) is directed by a PC software. Initially, the test is conducted by setting up frequency, acceleration as well as time elements through the software’s control settings. The settings are managed through Crystal Instrument’s engineering and data management (EDM) vibration control system (VCS) software. The preset commands of the test are then transferred into a vibration controller. The controller sends a drive signal to a power amplifier in order to drive a rectilinear shaker table. The shaker table (VT-500 by SENTEK DYNAMICS) transforms the drive signal into mechanical vibrations that are transferred into the energy harvester-sensor unit attached to the shaker armature. 

When performing the experiments, the lower end of the energy harvester-sensor unit is secured on top of the shaker table as shown in Figure 12. The vibration response from the energy harvester is measured by an accelerometer (PCB333B30 by PCB Piezotronics). The RF transmitter board situated on the static outer rim of the shaker table is connected to the energy harvester-sensor unit as shown in Figure 12a. The RF receiver circuit board is connected to the laptop via the USB to serial TTL level FTDI cable for live frequency response analysis as shown in Figure 12a,b.

## 5. Results and Discussion

**Figure 13:**
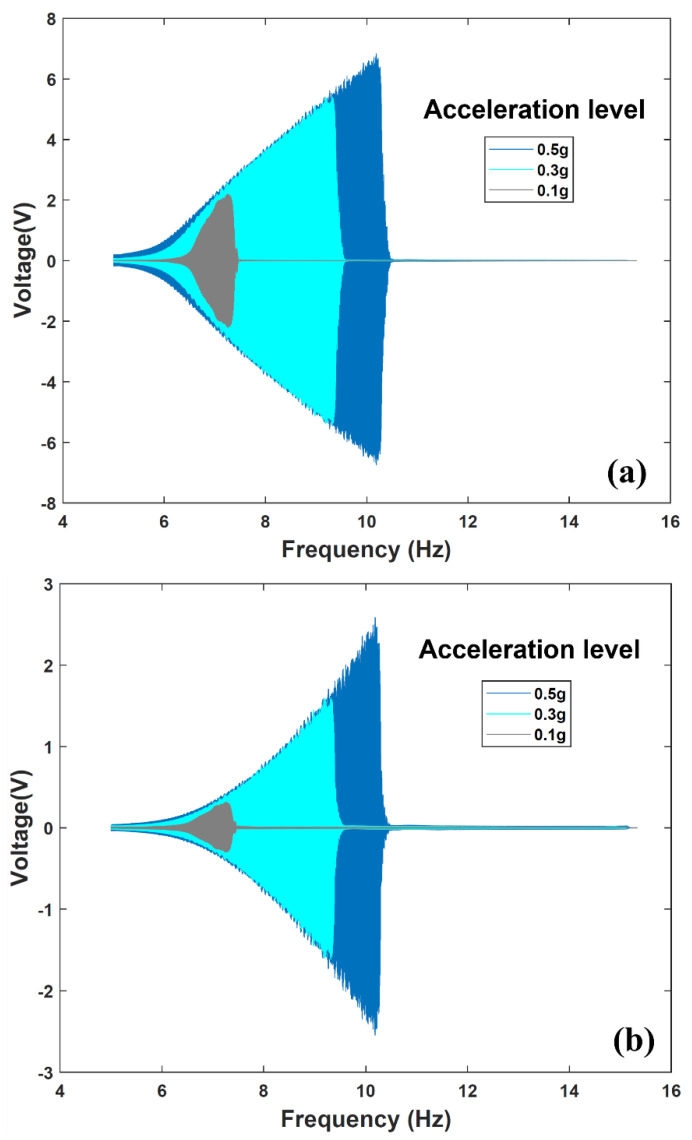
Energy harvester-sensor unit open circuit frequency response at a range of input accelerations. (**a**) Open circuit voltage of the energy harvester. (**b**) Open circuit voltage from the FR4 sensor diaphragm.

**Figure 14:**
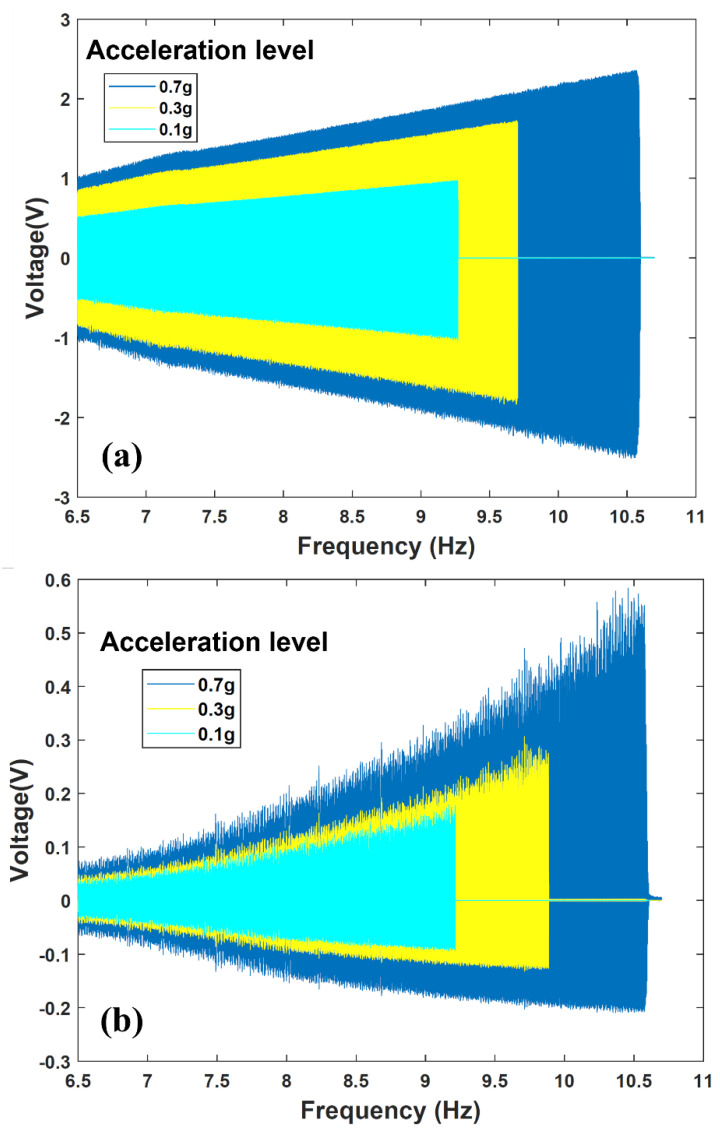
Energy harvester-sensor unit closed circuit frequency response at a range of input accelerations. (**a**) Energy harvester closed circuit voltage values measured across the microcontroller subsystem. (**b**) FR4 sensor diaphragm closed circuit voltage values measured across the input to the microcontroller.

Using the experimental apparatus shown in Figure 12, dynamic characterization of the fabricated energy harvester-sensor unit was performed. The resulting voltage frequency responses of both the energy harvester and sensor are shown in Figure 13 and Figure 14. The energy harvester-sensor unit was subject to fixed input acceleration values ranging from 0.1 g up to 0.7 g [m/s^2^] while the frequency was swept at a rate of 0.0833 Hz/s.

The nonlinear magnetic spring stiffness nature of the energy harvester is evident in the voltage frequency response as shown in both Figure 13 and Figure 14. That is, the repulsive magnetic forces experienced by the levitated magnet can be described as a nonlinear spring force [7,8]. This results in a hardening effect that is evident when comparing the trend in output voltage peaks shown in Figure 13 and Figure 14. One can notice that these peaks are shifting to higher frequencies as the input acceleration is increased. The nonlinear behavior of magnetic levitation-based energy harvesting system was studied extensively in our prior work [5,7,8,29]. Furthermore, during the frequency sweep, an abrupt sharp decline of the energy harvester’s frequency response is evident. This drop, known as the frequency jump phenomenon [7,8], is attributed to the coexistence of multiple energy states at the frequency branch. The discontinuity in the response is a characteristic of magnetic levitation-based energy harvesting systems [5,29].

**Figure 15:**
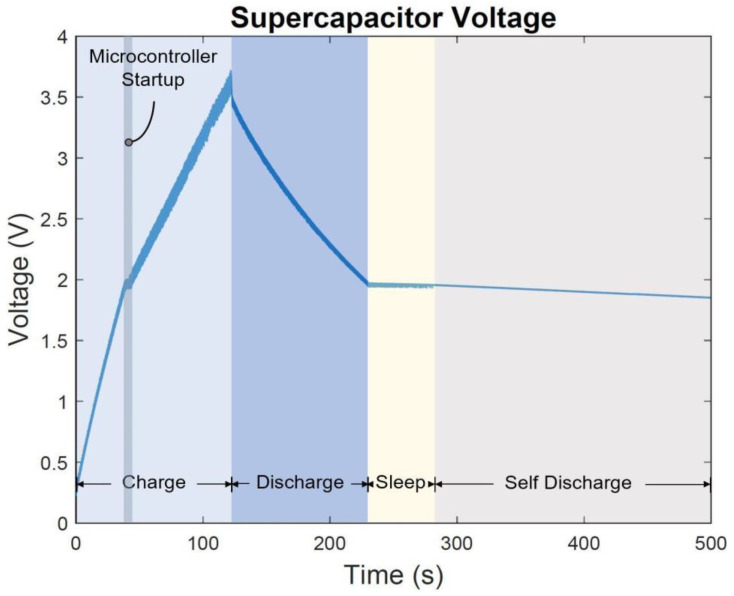
The supercapacitor voltage charge and discharge cycle during frequency sweep 6.5–15 Hz at 1 g [m/s^2^] acceleration level.

The supercapacitor charging and discharging cycles were measured using a data acquisition device (NI myDAQ) and the graphical programming environment LabVIEW software. The supercapacitor charging history is shown in Figure 15 where the maximum charge is held at 3.7 V after 122 s. During the charging cycle and after approximately 39 s, a slight shift in voltage level that lasts for 4 s occurs during the microcontroller waking up and voltage rails stabilization stage. The rate of the voltage held by the supercapacitor is seen to decrease from 44 mV/s to 22 mV/s after the microcontroller startup period. This is likely due to the fact that during this stage the system is draining the supercapacitor in order to operate. After approximately 122 s of charging, while simultaneously the energy harvester -sensor unit crosses the resonant frequency point during the frequency sweep, the supercapacitor discharging cycle starts to take effect at a voltage decline rate of 15 mV/s. The supercapacitor charging period after the microcontroller startup takes 79 s while it takes 108 s to discharge before the microcontroller enters the sleep mode as shown in Figure 15. At 230 s and 2 V, the microcontroller enters the sleep mode for a period that lasts approximately 50 s before the supercapacitor voltage level drops below 1.8 V which is no longer a sufficient amount of power for the microcontroller to operate. Consequently, the supercapacitor starts a self-discharge process as shown in Figure 15.

Next, the fully integrated, self-powered, self-contained sensor system was subject to harmonic oscillations and was tested under lab-controlled settings using the experimental apparatus shown in Figure 12. The resulting energy consumptions at different system states are shown in Table 2. The microcontroller internal oscillator was set to the lowest clock frequency of 32 kHz to minimize energy consumption. The ADC peripheral was configured to use the internal system clock oscillator. The microcontroller takes 10 measurements from the sensor signal during analog to digital bit conversion at a sampling rate of 51 kHz. After the measurements conversion is completed in 19.55 µs, the data are stored in the 16-bit ADC results register. The data are then retrieved by the EUSART serial communication peripheral, and the 10-bit data frame is transmitted asynchronously with 1 start bit and 1 stop bit added to each 8-bit sensor data packet and no parity bit. 

**Figure 16:**
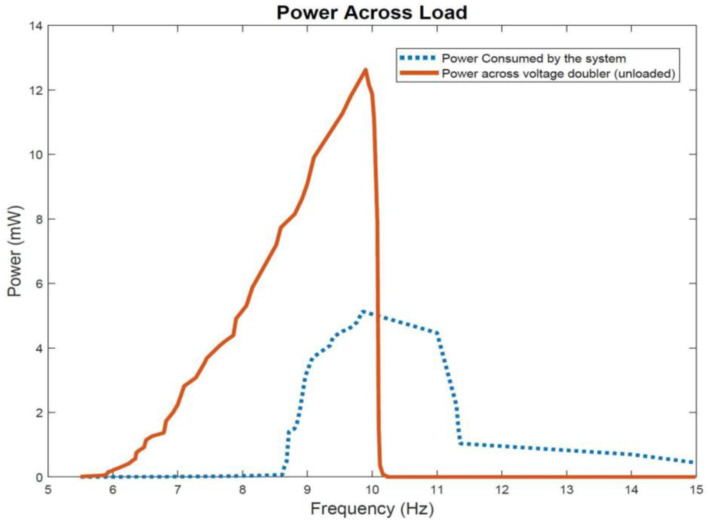
Power generated from the energy harvester at an unloaded voltage doubler output compared to power consumed by the microcontroller-transmitter subsystem load.

Figure 16 shows the power consumed by the transmitter subsystem as well as the power generated at an unloaded voltage doubler circuit. The power generated from the unloaded voltage doubler at resonant frequency is 12.6 mW and it exceeds the power consumed by the transmitter subsystem load of 5.1 mW. In the power consumed curve, the decline in power is more gradual compared to the abrupt decline seen in the power generated curve due to having the supercapacitor in the transmitter subsystem circuitry. In both power curves, the profiles show maximum power generation and consumption at resonant frequency of 9.9 Hz.

The operation and realization of the fully integrated self-powered fault detection system are examined next. A video recording of the self-powered, self-contained, environment-friendly, and wireless vibration sensor during data acquisition and transmission process is included in the Supplementary Materials. The video recording includes segments from the custom developed fault detection app along with the EDM shaker table vibration control software and the energy harvester and sensor voltage waveforms as well as the experimental setup showing the fabricated energy harvester-sensor unit mounted on the shaker table.

**Figure 17:**
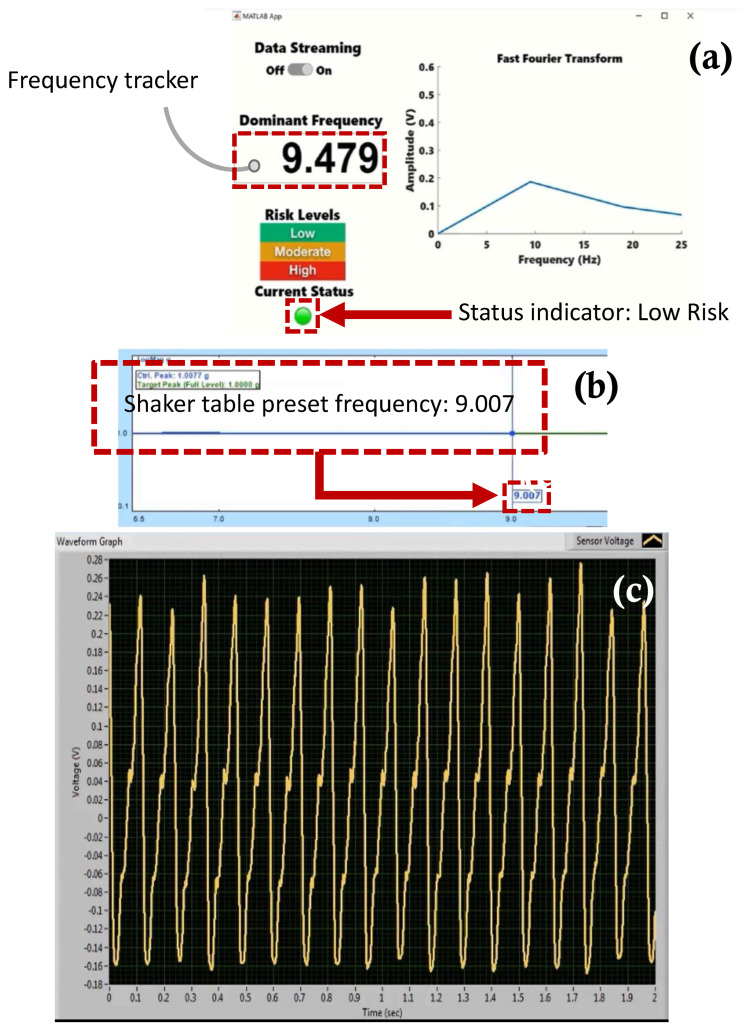
Screen captures of the self −powered, self −contained, vibration sensor system taken from the demonstration experiment: (**a**) custom developed fault detection app with a frequency tracker feature and status indicator for risk monitoring, (**b**) EDM shaker table vibration control software with preset frequency monitor, and (**c**) sensor voltage waveform monitor at preset frequency 9.007 Hz.

**Figure 18:**
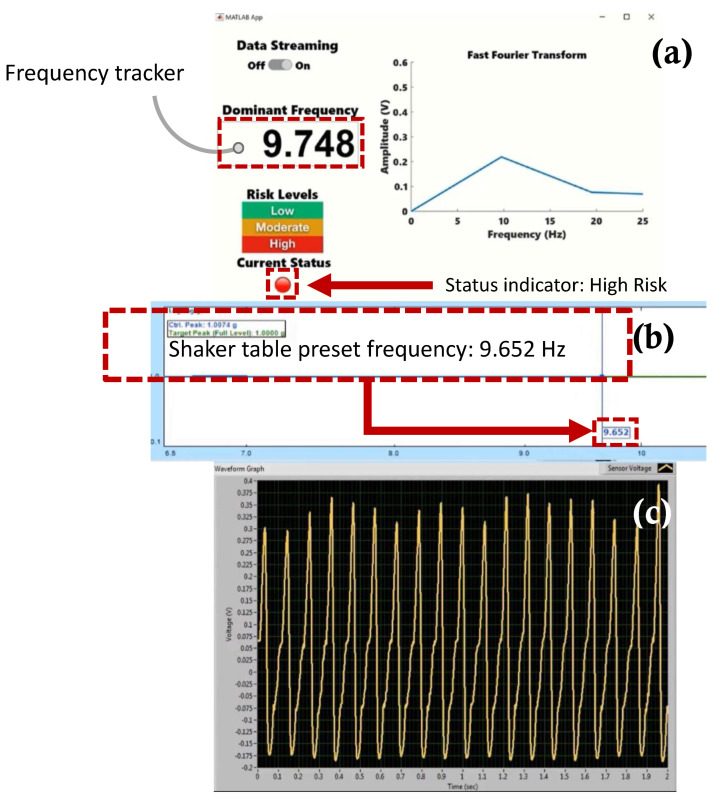
Screen captures of the self −powered, self −contained, vibration sensor system taken from the demonstration experiment: (**a**) custom developed fault detection app with a frequency tracker feature and status indicator for risk monitoring, (**b**) EDM shaker table vibration control software with preset frequency monitor, and (**c**) sensor voltage waveform monitor at preset frequency 9.652 Hz.

**Figure 19:**
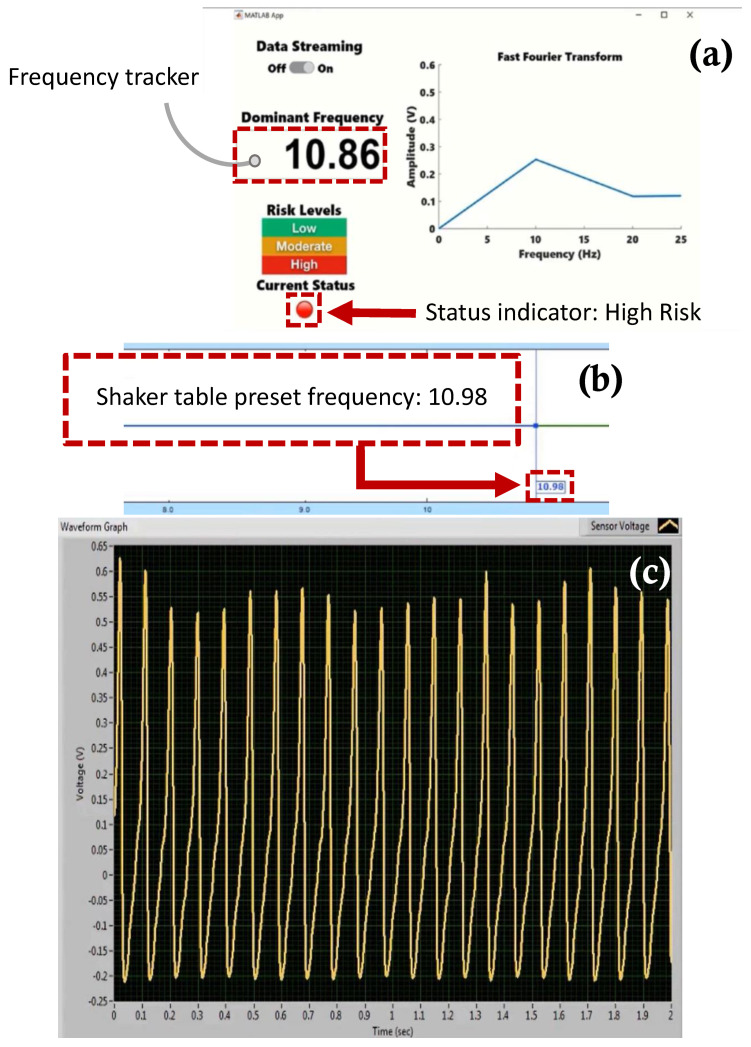
Screen captures of the self −powered, self −contained, vibration sensor system taken from the demonstration experiment: (**a**) custom developed fault detection app with a frequency tracker feature and status indicator for risk monitoring, (**b**) EDM shaker table vibration control software with preset frequency monitor, and (**c**) sensor voltage waveform monitor at preset frequency 10.98 Hz.

**Figure 20:**
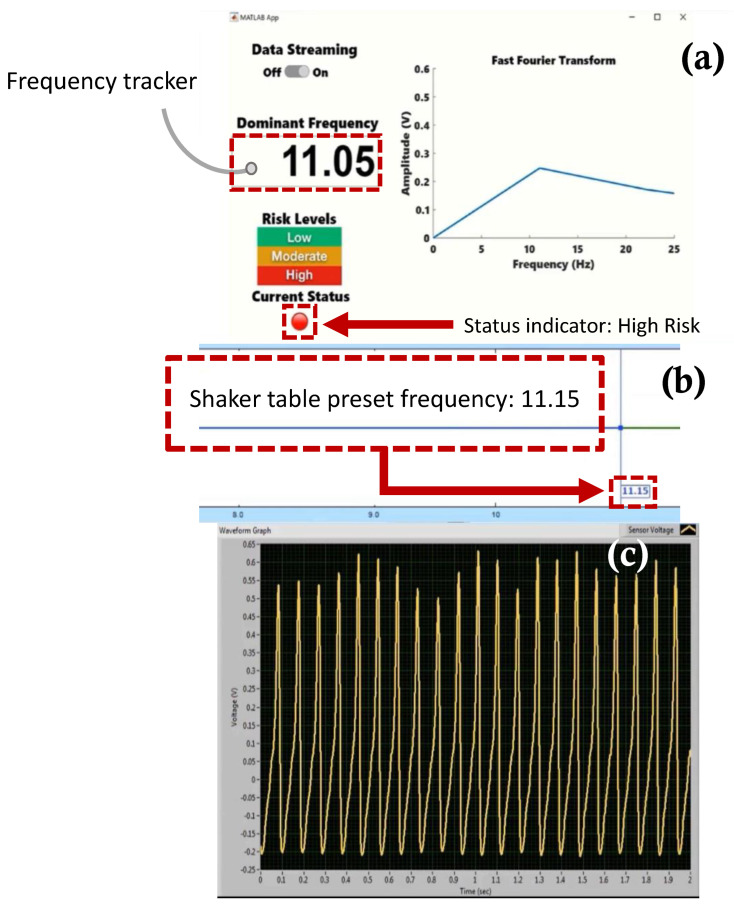
Screen captures of the self −powered, self −contained, vibration sensor system taken from the demonstration experiment: (**a**) custom developed fault detection app with a frequency tracker feature and status indicator for risk monitoring, (**b**) EDM shaker table vibration control software with preset frequency monitor, (**c**) sensor voltage waveform monitor at preset frequency 11.15 Hz.

Figure 17, Figure 18, Figure 19 and Figure 20 show selected timeframes showing the operation of the fully integrated self-powered wireless fault detection system at different stages during the system’s operation. Figure 17, Figure 18, Figure 19 and Figure 20 demonstrate the ability to self-power the sensor and the ability of the frequency tracker in the custom developed app to detect the vibration frequency from the self-powered sensor. For example, Figure 17a shows the vibration frequency from the sensor, detected by the frequency tracker in the custom developed app, at 9.479 Hz. The sensor vibration frequency value can be confirmed by the EDM software shaker table preset frequency, as shown in Figure 17b. Here, the preset frequency was approximately 9.007 Hz. Thus, there is a very slight discrepancy between the frequency detected by the frequency tracker in the custom developed app and the preset frequency from the shaker table. The error is estimated at approximately 5%. This discrepancy may be attributed to a few factors including the high noise in the AM transmitter/receiver module that is not filtered out during the FFT filtering process. Other factors include the use of the internal microcontroller oscillator in this work as opposed to using an external oscillator which would have resulted in a more accurate frequency stability. A bitrate estimated error of approximately 1.24% in the transmitted data is also a contributing factor to the overall error in the received and filtered data. Moreover, Figure 17c demonstrates the output signal from the fabricated self-powered, self-contained senor as the top magnet moves inside the top coil in response to the detected vibrations. That is, the dynamic displacement induced in the sensor causes induced voltage in the top coil surrounding the top guided magnet as shown in Figure 17c. Here, the power generated by the energy harvester is used to operate the RF transmitter circuit. The output voltage from the sensor is then sampled by the microcontroller on the transmitter circuit. The app, shown in Figure 17a, also demonstrates successful status indicator monitoring capabilities where it shows a green light, indicating a low level of acceleration experienced by the sensor (i.e., low risk operation). Similarly, Figure 18, Figure 19 and Figure 20 show subsequent timeframes from the demonstration experiment. In those timeframes, the sensor is self-powered, and the app detects a transition from a lower risk operation to a higher risk operation as seen signaled by the status indicator turning red in Figure 18. In Figure 18, the preset frequency from the vibration source (i.e., the shaker table) was approximately 9.652 Hz whereas the app detected tracker frequency was at 9.748 Hz. This results in a small error of approximately 1%. Likewise, the next timeframe in Figure 19 shows the preset frequency was at 10.98 Hz with the app detecting a 10.86 Hz also corresponding to a 1% error in the detected frequency value. The last presented timeframe from the demonstration experiment is shown in Figure 20 with the app detecting a frequency of 11.05 Hz compared to the preset frequency of 11.15 Hz resulting in only a 0.9% error. The mean absolute error value for all frequencies under investigation is found to be 0.8 while the mean percent error value is 2.6%.

## 6. Conclusions

In this work, we have introduced a novel self-powered self-contained wireless vibration sensor for fault detection in dynamic structures. The energy harvester-sensor unit is based on dual mass moving magnets. The voltages are extracted from the moving magnets by the coil surrounding the casing around the magnets. The power produced by the energy harvester subunit is used to operate an RF based transmitter subsystem. The transmitter subsystem sends mechanical vibration levels through a sensor subunit to a custom developed fault detection app wirelessly. The app notifies the user of the degree of risk associated with the operation by applying an FFT algorithm to the transmitted vibration data. The app can identify the frequency of the vibration with a low error of approximately 1% in most of the transmitted values. Unlike commonly studied self-powered vibration based WSN transmitter subsystems, this work utilizes the active power of the sensor subunit during the transmission process as opposed to requiring to power a passive vibration sensor. The transmitter subsystem operates at ultra-low power where the total consumption of energy to transmit a sensor value is approximately 0.894 µJ at 3 V. The transmitter subsystem can transmit the data from the sensor at a minimum operable acceleration of 0.7 g [m/s^2^] and an excitation range of 5–10.6 Hz. The significance in this work also lies in the low frequency required to operate the energy harvester-sensor unit that is widely available in many surrounding environments. Future work will consider employing a more energy-aware circuit with maximum power point (MPPT) tracking capability. This enhancement will allow for a self-sufficient stand-alone field operation of the energy harvester sensor unit. 

## Figures and Tables

**Table 1:** Specification and details of the fabricated energy harvester-sensor unit.

Specification	Value
**Energy Harvester**	
Coil turns number	450
Coil resistance	93 Ω
Minimum operable acceleration	0.7 g m/s^2^
Excitation frequency range	5–10.6 Hz
Casing material	3D printed polylactic acid (PLA)
Coil type	40 AWG enameled copper
Magnet’s material	NdFeB type N42
**Sensor**	
Coil turns number	1500
Coil resistance	890 Ω
Spring diaphragm material	FR4 glass-reinforced epoxy resin laminate
Coil type	40 AWG enameled copper
Magnet’s material	NdFeB type N42

**Table 2:** Transmitter circuit subsystem energy consumption at different program states.

Component	Power	Duration	Energy
Microcontroller startup (32 kHz)	0.879 mW	22.5 µs	19.7 nJ
Microcontroller operation (32 kHz)	0.756 mW	17.6 µs	13.32 nJ
Radio transmission (8 bits, 76.8 kbps)	8.58 mW	0.1 ms	0.858 µJ
Sensor reading	0.144 mW	19.55 µs	2.8 nJ
Total			0.894 µJ

## Data Availability

Data are contained within the article or Supplementary Material. The data presented in this study are available in [this manuscript].

## Supplementary Materials

The following supporting information can be downloaded at: https://www.mdpi.com/article/10.3390/s22062352/s1. Video S1: Demonstration experiment.
